# A Potentially Fatal Cause of Sciatica: A Rare Case of Nerve Compression Caused by a Pseudoaneurysm of the Renal Artery of a Transplanted Kidney

**DOI:** 10.7759/cureus.66130

**Published:** 2024-08-04

**Authors:** Andreia Leal, Mariana Magalhães, Nuno Teles Pinto

**Affiliations:** 1 Family Medicine, Unidade Local de Saúde de Santo António, Porto, PRT

**Keywords:** focal segmental glomerular sclerosis, nerve compression, vascular complications, pseudoaneurysm, kidney transplantation

## Abstract

Renal transplant is the gold standard treatment for end-stage kidney disease (ESKD). Despite the evolution of renal transplant procedures, complications can still occur. Transplant renal artery pseudoaneurysm is a rare but potentially life-threatening complication, which can be asymptomatic or cause mass-effect symptoms. We report an unusual case of a pseudoaneurysm of an unfunctional renal transplant that caused a femoral nerve compression, mimicking lumbosacral radiculopathy. The case concerns a 38-year-old woman with primary focal segmental glomerulosclerosis (FSGS) that progressed to ESKD. The patient underwent a kidney transplant that failed a few years after the surgery. More than 10 years later, she presented with symptoms consistent with lumbosacral radiculopathy, which was ultimately diagnosed as femoral nerve compression caused by a transplant renal artery pseudoaneurysm. This case emphasizes that each patient’s medical history should always be considered when assessing even common complaints because rare causes can manifest in frequent symptoms. On the other hand, this case makes us reflect on weighing up the cost/benefit of some diagnostic investigations, as it is important not only to investigate the most common causes but also to rule out, in selected patients, those that, although rare, can be life-threatening.

## Introduction

Renal transplant is considered the gold standard treatment for end-stage kidney disease (ESKD) having the potential to reduce mortality and improve quality of life in comparison to chronic dialysis. The advances in immunosuppression, surgical technique, and protocol-driven management of transplant recipients have made renal transplant a safe procedure with durable long-term outcomes [1]. Despite the evolution of renal transplant procedures, complications can still occur. Vascular complications occur in 3-15% of kidney transplant patients, with transplant renal artery stenosis being the most common, but also including renal vein or artery thrombosis, hematoma, and pseudoaneurysm formation [2,3]. Transplant renal artery pseudoaneurysm is a rare complication, occurring in less than 1% of cases [4]. It can be asymptomatic or cause symptoms due to renal failure, mass effect, or associated infection, and it is potentially life-threatening as a sudden rupture can lead to severe hemorrhage [5]. Understanding these potential complications is crucial for postoperative care and long-term management of renal transplant patients.

Focal segmental glomerulosclerosis (FSGS) is one of the conditions that progresses to ESKD. It is a histologic lesion, rather than a specific disease entity, characterized by the presence of scattered (segmental) sclerosis in some (focal) glomeruli observed under light microscopy on a kidney biopsy [6]. It is commonly associated with nephrotic syndrome, and the primary treatment goal is to achieve remission of proteinuria, using glucocorticoids and calcineurin inhibitors [7], along with supportive measures, such as renin-angiotensin inhibition. However, FSGS commonly progresses to ESKD [8], and in cases of kidney transplantation, it may recur in the transplanted kidney [9]. Patients with histology evidence of FSGS receiving kidney grafts have a significantly inferior five-year graft survival compared to transplant recipients with any other cause of kidney disease (81 vs. 88%) [10].

We report a case of a 38-year-old woman with primary FSGS that progressed to ESKD. The patient underwent a kidney transplant that failed a few years after the surgery. More than 10 years later, she presented with symptoms consistent with lumbosacral radiculopathy, which was ultimately diagnosed as nerve compression caused by a transplant renal artery pseudoaneurysm.

## Case presentation

We describe a case of a 38-year-old Caucasian woman, non-smoker, with a BMI of 22 kg/m^2^ and a chronic history of FSGS, with secondary arterial hypertension, that led to a kidney transplant. Diagnosed with FSGS in 1998, at the age of 12, she subsequently developed cortico-resistance and progressive renal failure. Renal replacement therapy (RRT) using hemodialysis began in January 2003 and then switched to peritoneal dialysis in April 2003. At the age of 22 (November 2008), a kidney transplant from a cadaver donor was performed, with no perioperative complications, with immediate graft function. However, three months later, a recurrence of FSGS was detected in a transplant biopsy, after the beginning of nephrotic proteinuria and progressive serum albumin reduction. Over the following years, she was hospitalized several times in the context of calcineurin inhibitor toxicity, graft pyelonephritis, and resistant hypertension. Graft lithiasis with hydronephrosis was detected and renal function progressively worsened, leading to graft loss in August 2012 (3.5 years after transplant). Hemodialysis was therefore restarted, and the non-functioning graft remained in the left iliac fossa (LIF). The patient remained clinically stable, undergoing hemodialysis three times a week and medicated with sevelamer carbonate 2.4g qid; calcium acetate 435 mg + magnesium carbonate 235 mg, two tabs tid; cinacalcet hydrochloride 30 mg twice a week; carvedilol 12.5 mg bid; amlodipine 5 mg bid; pantoprazole 20 mg qd; and alprazolam 1 mg qd.

In December 2023, she complained of a nonspecific pain in the left lower limb that she had been feeling for one week, with no other accompanying symptoms and no history of a fall or trauma. There were no abnormal findings on musculoskeletal and neurological examination of the limbs; abdominal examination was not performed. She was discharged with a combination of paracetamol 325 mg and tramadol 37.5 mg tid for pain management. In February 2024, at an appointment with her family doctor, she reported continued and worsening pain, with no relief from the prescribed analgesia. The pain radiated from the left lumbar region with dysesthesia, a burning sensation, and paresthesia from the anterior left thigh to the lower knee. She also reported left claudication due to decreased muscle strength. On objective examination directed to the left lower limb, she presented gait claudication and atrophy of the rectus femoris, absence of the patellar reflex, decreased skin sensitivity in the anterior region of the thigh, and decreased muscle strength when extending and flexing the knee and flexing the thigh. In the suspicion of radiculopathy, the family doctor requested a lumbar X-ray, which showed no abnormalities, and a lumbar computerized tomography (CT), which revealed no signs of radicular compromise and a space-occupying lesion (7 x 10 cm) on the iliac bone and homonymous muscle, partially calcified (apparently the renal graft). In view of these findings, the doctor requested a Neurology consultation at the referral hospital.

**Figure 1:**
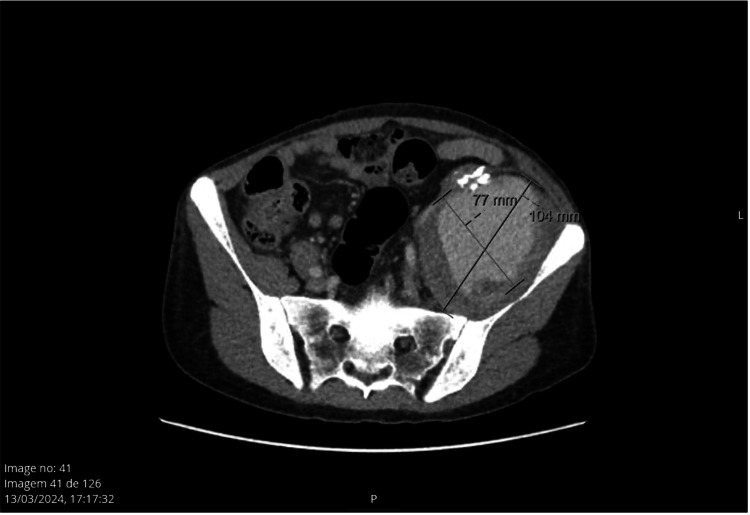
CT scan showing a mass of 77 x 104 mm in the left iliac fossa.

In March 2024, while still waiting for the Neurology appointment, the patient went to the emergency department (ER) due to worsening pain and extension deficit in the left knee, which had been going on for two days. Objectively, the patient displayed impaired extension of the left knee (grade 2/5) on the dependency of the L3/femoral nerve and pulsatile but hardened swelling in the LIF. All pulses in the lower limbs were palpable. X-rays and lumbar CT scans were performed, and their results showed that the mass in the LIF (Figure 1) was with high probability responsible for the compression of the femoral neurovascular axis, justifying the neurological deficit and pain.

**Figure 2:**
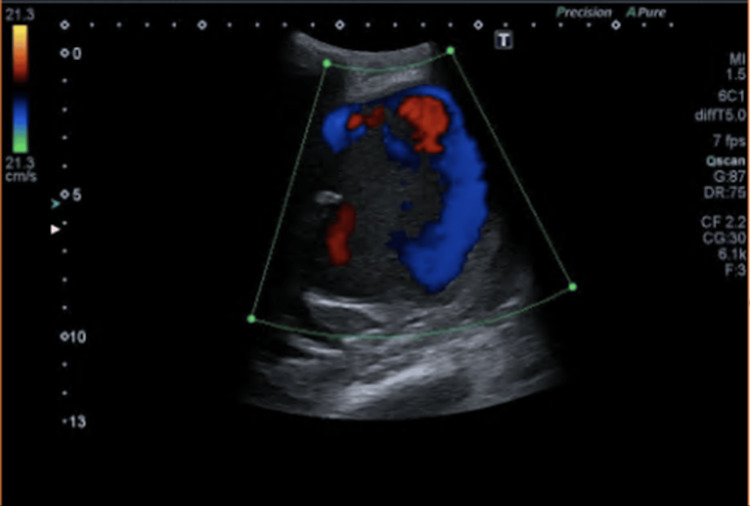
Abdominal-pelvic Doppler ultrasound showing a yin-yang sign.

An abdominal-pelvic ultrasound was performed, which showed, in correspondence with the voluminous heterogeneous mass mentioned on the CT, a well-defined image, with mobile, pulsatile echogenic content on the color Doppler study - yin-yang sign - compatible with the diagnosis of pseudoaneurysm of the previous renal graft (Figure 2). 

**Figure 3:**
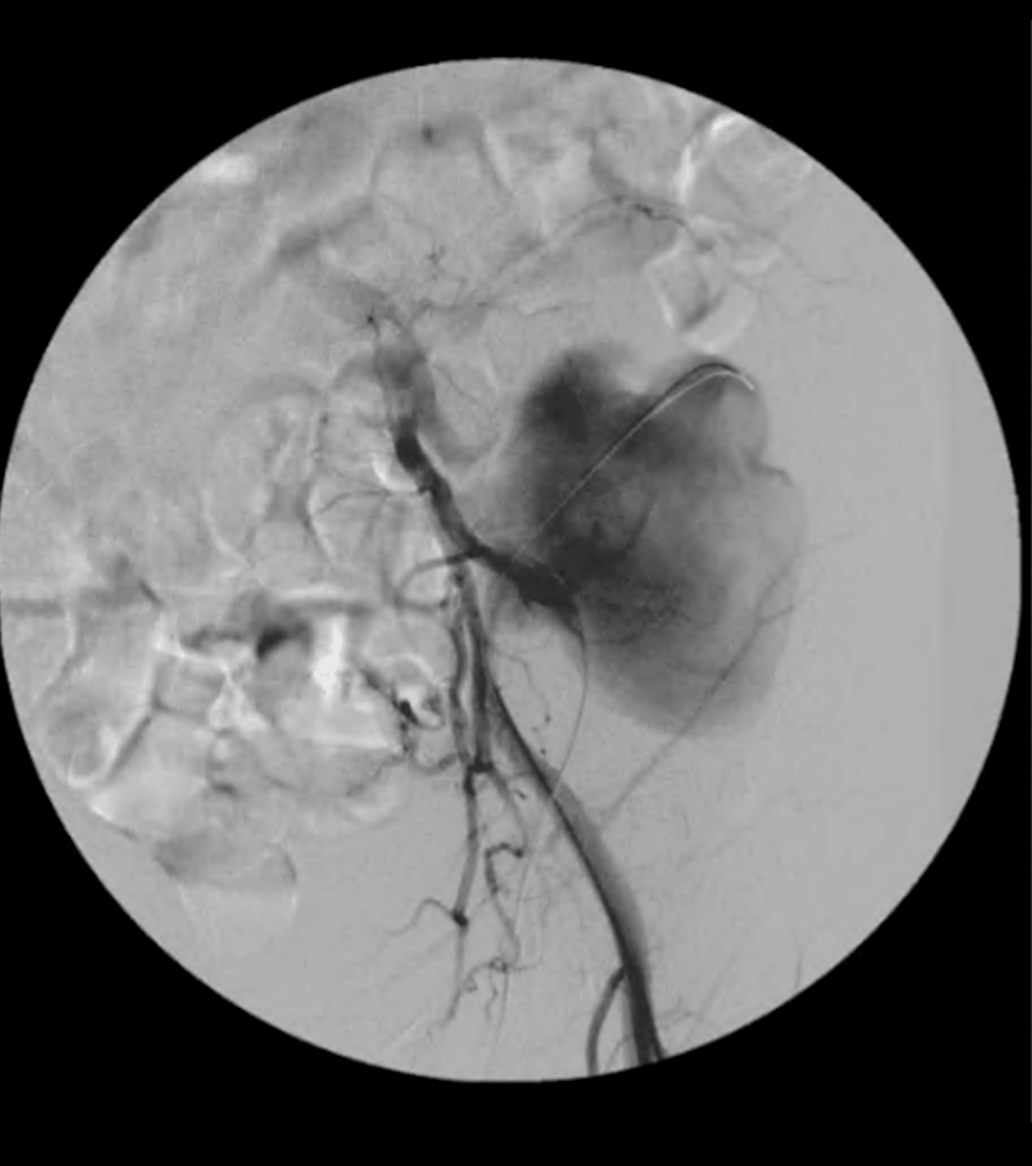
Angiography performed during vascular surgery showing the pseudoaneurysm of the renal graft.

**Figure 4:**
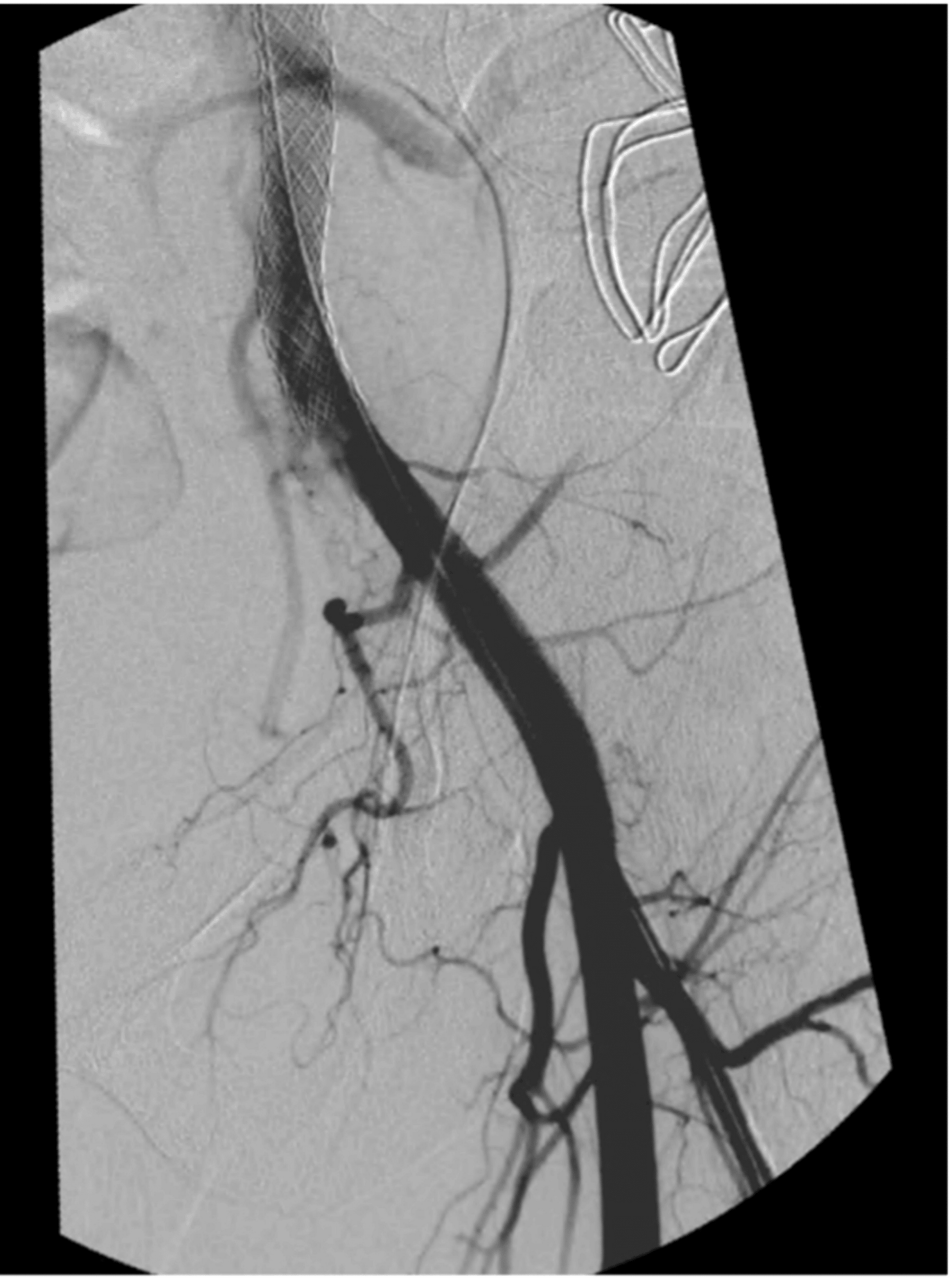
Angiography performed after stent-graft occlusion showing a complete resolution of the pseudoaneurysm.

The patient underwent urgent endovascular surgery, for stent-graft occlusion of the arterial anastomosis of the transplanted kidney, percutaneous drainage of the hematic collection of the false aneurysm (Figure 3), and embolization with coils of the left deep circumflex iliac artery (Figure 4). The surgical procedure and the postoperative period were uneventful, and the patient was discharged with almost total resolution of the pain complaints and with follow-up future appointments with Vascular Surgery and Neurology. 

In May 2024, a control CT angiogram showed a total resolution of the pseudoaneurysm, with no areas suggestive of contrast leakage and a slight reduction in size to around 8.1 x 7.5 cm. At the end of May 2024, the patient had the Neurology appointment (previously requested by the family doctor), showing significant improvement: muscle strength gradually improved, with no gait claudication, maintaining paresthesia and sporadic neuralgic stabbing-like pain in the anterior aspect of her left thigh, which she tolerated. On neurological examination, there were no sensory or motor deficits, but an abolished left patellar osteo-tendon reflex and left quadriceps muscle atrophy were still detected. The patient currently awaits electromyography (EMG) to assess the neurophysiological damage caused by the compressive injury to the femoral nerve.

## Discussion

Osteoarticular and musculoskeletal complaints are among the most frequent reasons for seeking acute illness consultation in primary health care [11] and are therefore a common situation in the clinical practice of family doctors. Pain in a lower limb is mostly of musculoskeletal or neuropathic origin. The signs that should raise suspicion of radiculopathy include specific pain characteristics - tingling, burning, or shock sensation that can radiate along the nerve path - and segmental sensory-motor deficits - paresthesia and declining sensation for temperature, touch, vibration, or proprioception [12]. The corresponding deep segmental tendon reflexes may be diminished or absent, and in more advanced stages, the muscles innervated by the affected motor root may become weak and atrophic and show flaccidity and fasciculations [12]. The most common etiologies of femoral neuropathy are compression (mostly vascular or neoplastic) and perioperative stretch [13].

Complications of renal transplants occur in about 15-17% of cases and can be classified as surgical - vascular, urologic, and wound infection - or pathological/nephrogenic - rejection, infectious, and cardiovascular [14]. A delay in appropriate diagnosis and treatment of any of these complications raises morbidity and mortality [15]. Regarding vascular complications, pseudoaneurysms can be intra or extrarenal. The first type is most common and usually resolves spontaneously with time. On the other hand, extrarenal pseudoaneurysms (EPSAs) have an incidence rate of less than 1% and usually need endovascular or open surgical repair [16]. They can be diagnosed days or years after the transplant and their clinical presentation varies considerably, from totally asymptomatic to a rupture that can lead to a life-threatening hemorrhage. Other symptoms include abdominal pain, a pulsatile mass, lower limb ischemia, allograft dysfunction, anemia, hypertension, or signs of infection [4]. Doppler ultrasonography is the first diagnostic tool used, indicating turbulent arterial and venous blood mixing (ideally showing the pathognomonic yin-yang sign). Magnetic resonance (MR) or CT angiography can confirm the diagnosis and evaluate if it affects surrounding structures [17]. Conventional angiography shows the pseudoaneurysm’s precise location and permits a percutaneous approach, as it was performed in the presented case.

Although pseudoaneurysms can be one of the reasons for graft loss [1-5], in this case, the appearance of this complication followed the graft failure and did not constitute its cause. The patient, who had been clinically stable for the last 10 years with a non-functioning kidney graft, presented with complaints compatible with a relatively rapidly progressing radiculopathy. Her family doctor directed the study to the suspected diagnosis of radiculopathy, looking for alterations at the vertebral level through a lumbar CT scan, which was a right assumption. However, considering the patient’s kidney graft in the LIF and her complaints in the left lower limb, the possible vascular complications of the kidney graft should not have been overlooked, as, although rare, they can be life-threatening. Therefore, it would be important to perform a Doppler ultrasound to rule out a nerve compression due to a possible false aneurysm, considering that it is a non-expensive exam that would help to make the correct diagnosis and to drive the treatment in the correct direction.

## Conclusions

We report an unusual case of a pseudoaneurysm of an unfunctional renal transplant that caused a femoral nerve compression, mimicking lumbosacral radiculopathy. This case emphasizes that each patient's medical history should always be considered when assessing even common complaints because rare causes can manifest in frequent symptoms. On the other hand, it makes us reflect on weighing up the cost/benefit ratio of some diagnostic investigations. It is important, in the initial approach, not only to investigate the most common causes but also to rule out less frequent possible causes in selected patients, which, although rare, put patients' lives at risk.

Owing to advanced surgical techniques, kidney transplantation continues to be a safe treatment modality. However, knowing the incidence and clinical manifestations of potential complications is necessary for early detection and treatment. In this case, it would be important to maintain Doppler ultrasound monitoring of the graft, even after loss of function. It was not the family doctor’s job to have such specific knowledge, but some communication between colleagues could have helped. This can be a wake-up call to the importance of the various medical and surgical specialties that work with the same patient to provide some essential information to each other, including the patients’ family doctors, who are the ones who most frequently and for the longest time accompany them.
